# What you see is what you breathe? Estimating air pollution spatial variation using street level imagery

**DOI:** 10.3390/rs14143429

**Published:** 2022-07-17

**Authors:** Esra Suel, Meytar Sorek-Hamer, Izabela Moise, Michael von Pohle, Adwait Sahasrabhojanee, Ata Akbari Asanjan, Raphael E. Arku, Abosede S Alli, Benjamin Barratt, Sierra N Clark, Ariane Middel, Emily Deardorff, Violet Lingenfelter, Nikunj Oza, Nishant Yadav, Majid Ezzati, Michael Brauer

**Affiliations:** 1 Imperial College London; 2 ETH Zurich; 3 Universities Space Research Association (USRA); 4 NASA Ames Research Center; 5 University of Massachusetts Amherst; 6 Arizona State University; 7 San Diego State University; 8 UC Berkeley; 9 University of Ghana; 10 University of British Columbia; 11 University of Washington

**Keywords:** Computer vision, deep learning, street images, air pollution, data science, transferability, urban pollution

## Abstract

High spatial resolution information on urban air pollution levels is unavailable in many areas globally, partially due to high input data needs of existing estimation approaches. Here we introduce a computer vision method to estimate annual means for air pollution levels from street level images. We used annual mean estimates of NO_2_ and PM_2.5_ concentrations from locally calibrated models as labels from London, New York, and Vancouver to allow for compilation of a sufficiently large dataset (~250k images for each city). Our experimental setup is designed to quantify intra and intercity transferability of image-based model estimates. Performances were high and comparable to traditional land-use regression (LUR) and dispersion models when training and testing on images from the same city (R^2^ values between 0.51 and 0.95 when validated on data from ground monitoring stations). Like LUR models, transferability of models between cities in different geographies is more difficult. Specifically, transferability between the three cities i.e., London, New York, and Vancouver, which have similar pollution source profiles were moderately successful (R^2^ values between zero and 0.67). Comparatively, performances when transferring models trained on these cities with very different source profiles i.e., Accra in Ghana and Hong Kong were lower (R^2^ between zero and 0.21) suggesting the need for local calibration with local calibration using additional measurement data from cities that share similar source profiles.

## 1 Introduction

Air pollution has significant and widely accepted implications on health outcomes [1]–[4]. Advances in spatial estimation of air pollution concentrations have been instrumental both in establishing health effects and impact on the burden of disease [5], [6]. Yet, we currently lack information on air pollution levels in many areas globally [7] especially at sufficiently high spatial resolution to inform local planning and air quality management strategies. Limited spatial and temporal resolution of air quality models partially results from input data requirements of existing estimation approaches including physically based e.g., dispersion models, and geostatistical e.g., land use regression (LUR) models [8]. High quality air pollution measurements from ground measurement campaigns are required for development and calibration of city-wide air pollution models, yet they are very costly to implement and therefore not available for most cities around the world. Advances in deep learning methods and their success in computer vision applications has led to a growing interest in using images for estimating air pollution levels [8]–[16]. The rationale behind this interest is that information on pollution sources and common predictor variables used in traditional approaches (e.g., land use, traffic, built and natural environment features [17]) is, at least partially, visible from street level and satellite images. If utilized effectively, this approach has the potential for reducing input data requirements and scaling up to global coverage at low cost.

Increasing availability of images in cities makes feasible the extraction of visual information at high spatial resolution. In contrast, spatially detailed air pollutant concentration levels are typically not readily available. This is a substantial challenge for application of deep learning methods as they require very large numbers of labeled data (i.e., images and their corresponding air pollution levels) for training. One proposed solution [9], [13], [16], [18] is to collect images when conducting air pollution monitoring campaigns. However, such data are rarely available and costly to collect at the scale necessary for generalizing to unseen locations. A second approach is to extract intermediate user defined features from images that are known to be important predictors of air pollution (e.g., trees, buildings, cars, buses, roads) and to use them as explanatory variables to estimate pollutant levels with traditional regression methods [16]. This process, however, requires separate training on databases that may not contain crucial categories of interest or be publicly available. Further, deep learning methods that automatically learn relevant features commonly outperform methods based on hand-crafted features in visual recognition tasks [19]. In this study, we propose a third approach, to estimate pollution levels from raw images without extracting intermediate user defined features. As the numbers of monitoring locations (in hundreds) from ground measurement campaigns are insufficient for training deep learning models, we propose to use annual mean estimates of air pollutant concentrations from locally calibrated models as labels for training. These local models provide continuous surfaces of pollution estimates at high spatial resolution. Use of LUR or dispersion-based estimates matched with street level images allows for compilation of a large dataset of image-label pairs required for training deep learning models. Different types of images (e.g., street-level, aerial, and satellite images) will contain visual information captured from different viewpoints. Local, especially near-road, information (e.g., cars, building heights, built and natural environmental features) is more visible from street-images, while high resolution satellite and aerial images will contain more information on the spatial context (e.g., proximity to parks, forests, ocean that are not necessarily visible from street-level) [20]. Here, we use street-level images only to test whether locally rich information can be used to estimate spatial variation at high-resolution. Variation in NO_2_ is higher and influenced more by local sources likely visible from street images. PM_2.5_, on the other hand, is influenced by regional levels and secondary production which is less likely visible from street-images. LUR models, for NO_2_ especially, indicate that the variables describing the near-road environment explain much of the spatial variability within urban areas and we hypothesize that street-level imagery would be useful in NO_2_ modelling but less relevant for estimation of variability in PM_2.5_. However, even for PM_2.5_ while overall variability is harder to explain, some is explained by near-road predictors, and we hypothesize that imagery may provide a richer dataset for estimation than geospatial predictors that had been identified a prior.

Here, we describe the application of a deep learning approach to estimate air pollution spatial variation using street level images for PM_2.5_ and NO_2_. Our deep learning models work by automatically learning visual features associated with different levels of pollution to describe variation over space. These features may correspond to sources of pollution or pollution haze when visible from street images. First, we test how transferable i.e., similar these visual features are within (intracity) and across (intercity) cities where we have good data from city-wide models and ground monitoring stations. For training the models, we used annual mean NO_2_ and PM_2.5_ estimates from three cities in three different countries selected for their varying levels of pollution and the availability of high resolution concentration estimates: London (UK), New York (USA), and Vancouver (Canada) [21]–[26]. A disadvantage of this approach, however, is that the performance of the image-based models will be influenced by the performance of the source models whose outputs are treated as the truth during training phase. Here, we used previously published models from all three cities; we include their performances against measured concentrations. Second, we make estimates for two additional large cities: Accra (Ghana) and Hong Kong (PRC), as examples of locations where spatially resolved ground measurements are available. In Accra and Hong Kong, our estimates were based merely on street level images with no additional information on the spatial context (e.g., regional pollution levels or proximity to parks, ocean etc.). Our selection of cities from different geographies, countries, and source profiles is deliberate to make the task of transferability particularly difficult to better understand transferability capabilities of image based models in three different settings that vary in their level of expected difficulty for transferability performance: within the same-city and country, across cities with similar source profiles but different countries and geographies, and across cities with different source profiles and different geographies.

## 2 Materials and Methods

### 2.1 Land use regression and dispersion model outputs

**Figure 1:**
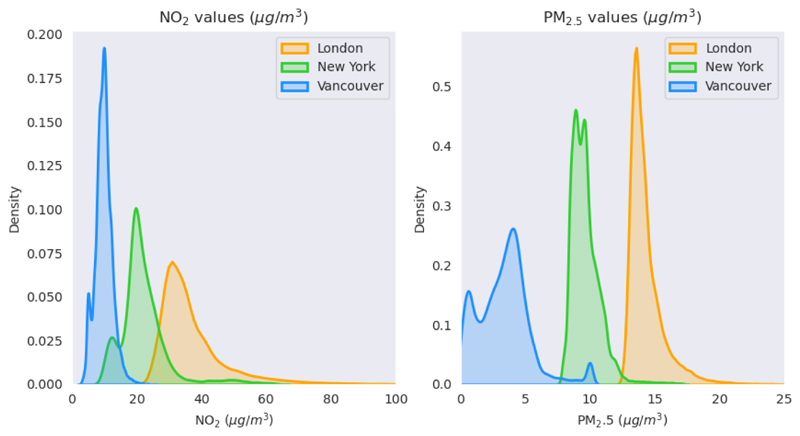
Distributions of annual mean NO_2_ and PM_2.5_ estimates from local land use regression and dispersion models. Figures show a clear distinction of air pollutant levels between the three study cities. These values are used as labels when training deep learning models using street level images.

**Figure 2:**
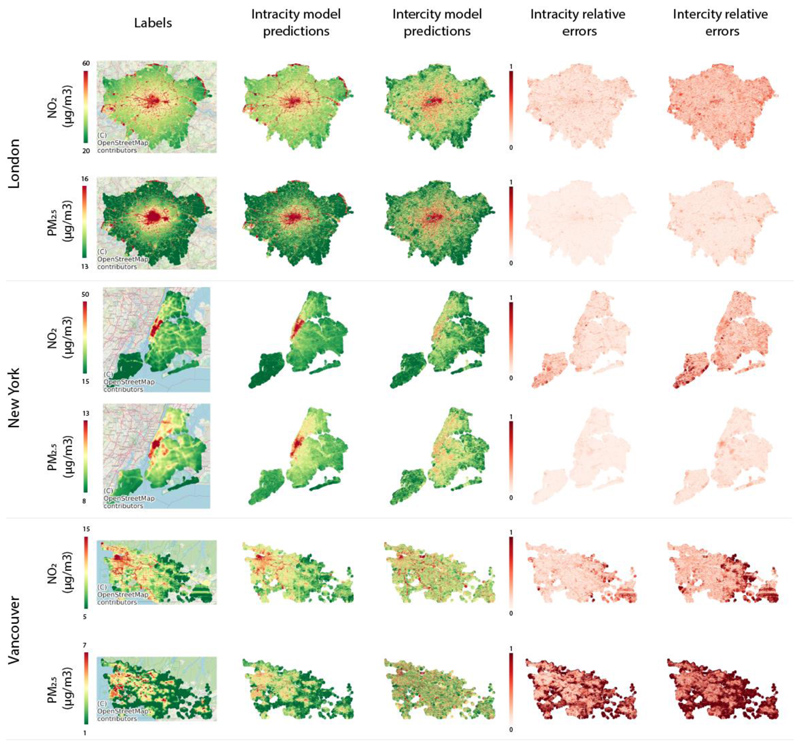
Comparison of labels and prediction maps of mean NO_2_ and PM_2.5_ levels for London, New York, and Vancouver. The label column displays the base map and the extent of the shapefiles used. We adjusted the color bar scale to visualize the spatial patterns for each city and pollutant where red areas correspond to high pollution and green to low pollution. Intracity model predictions show results from experiments where networks were trained and tested on images from the same city using four-fold cross validation. Intercity model predictions where the models were trained on two cities and tested on the third hold out city. Relative errors (absolute error divided by the magnitude of the label value) are also displayed where darker red colors indicate higher error rates.

We obtained annual mean NO_2_ (200m resolution raster) and PM_2.5_ (100m resolution raster) estimates for 2010 from three cities: London, New York, and Vancouver using existing most recent local models developed for these cities [21]–[28]. Land use regression (LUR) models were available for New York and Vancouver and an atmospheric dispersion model for London. Land use regression and dispersion models are the two main model types used for estimating spatial variability, and we selected three cities where we had access to images and high-resolution annual estimates of both pollutants. A dispersion model is available that is maintained to which we had access whereas there is no recent LUR for London for both pollutants. For other cities, LUR models were used and were available to us. The New York LUR models (r=0.69 for PM_2.5_ and r=0.79 for NO_2_ computed using ground measurements) included traffic density, proximity to bus routes, percentage of impervious surface, interior building space, proximity to industrial sites, proximity to heat and hot water boilers as geospatial predictor variables. For Vancouver (r=0.33 for PM_2.5_ and r=0.86 for NO_2_) the model included vehicle density, proximity to commercial areas, elevation, and population density as predictor variables. The model in London (r=0.65 for PM_2.5_ and r=0.49 for NO_2_) used distance to roads by type, street canyon type, and meteorological inputs as key input variables. Distributions of annual estimates for both pollutants were distinct and almost non-overlapping for each of the study cities (Figure 1). The differences in absolute levels of pollution across cities is by design for our experiments and makes intercity transferability challenging, as discussed in Section 3. NO_2_ estimates demonstrated a clear spatial trend following roads as the main source, PM_2.5_ are more heterogeneously spread reflecting the diversity of sources, the impact of regional factors and secondary production (Figures 2). Vancouver is characterized by low levels of NO_2_ and PM_2.5_ concentrations. Areas near downtown and the Vancouver International Airport show relatively higher levels of air pollution, whereas large park and mountainous areas have low pollution levels (Figure 2). This resulted in a bimodal distribution for both PM_2.5_ and NO_2_ in Vancouver with a distinct local peak at the lower end in Figure 1. A similar bimodal distribution is observed for New York especially for NO_2_, whereas London estimates showed unimodal distributions for both pollutants. Both New York and London are right skewed. In New York, densely populated Manhattan as well as Queens and Brooklyn Heights stand out with their high levels of pollution. In London, similarly, the densely populated city center as well as areas near the major roads have high levels of pollution. London contained the highest estimates for both pollutants.

### 2.2 Street level images

To obtain images, a 100m grid for London, New York, and Vancouver was created using the boundary shape files covering the model output domains. 100m grids for Accra and Hong Kong were created using the city boundary shape files. For each of the grid centroid points, we used the nearest street level panorama image location available from Google Street View that was captured from years 2008 to 2012 (+/- 2 years from the model year) for London, New York, and Vancouver. For each image location, four images of size 224x224 pixels were accessed specifying the camera direction (i.e., 0°, 90°, 180°, 270°) relative to the Street View vehicle to cover a 360° view. These images were then concatenated resulting in a single image of size 224x896 pixels used as input to the network as explained in Section 2.4. Nearly 820,000 street level images, (378,856 images for London, 198,300 for New York, and 242,248 for Vancouver) were matched with NO_2_ and PM_2.5_ model estimates (see Section 2.1) as outcome labels. For Accra and Hong Kong, we obtained the most recent panorama image for each of the grid centroid points corresponding to years 2018 to 2019 resulting in, respectively, 250,460 and 265,308 images in total.

### 2.3 Ground monitoring stations

Site measurement data were obtained from ground-based air monitoring stations from each of the three study cities for evaluating the image-based air pollution estimates. More information about stations and data collection protocols used are available from [23], [29]–[32]. Stations were included for evaluations where a street image (and consequently an image-based estimate) was available within 50 meters. For London, daily station data from [31] was used to calculate annual means of measured pollution over ground stations that had at least one measured value every month for 2010. For Vancouver and New York, annual means of measured data from [23], [29], [30], [32] were used for stations that had collected data for more than half of the year during 2010. We used a total of 59 ground NO_2_ sites with eight, four, and 47 sites in London, New York, and Vancouver respectively. For PM_2.5_, a total of 15 stations were used with nine, two, and four sites, respectively. London had the highest average pollutant values for NO_2_ and PM_2.5_ (63.4 and 16.3 µg/m^3^, respectively), followed by New York (54.1 and 11.3 µg/m^3^) and Vancouver with the lowest (11.0 and 4.0 µg/m^3^) levels. Unfortunately, the numbers of available ground monitoring stations were very low. More data from ground monitoring stations are needed for evaluations to generalize from our analysis from comparisons between image based predictions and ground truth measurements. We report our findings in the following section.

The metrics used for evaluations in Table 1 need to be interpreted carefully for cities with small numbers of ground station data. Ground monitoring station data offered an evaluation based upon a measured (ground truth) dataset in addition to the evaluations done on modelled data. Since the models developed for New York, Vancouver, and London were not calibrated using ground monitoring station data, these measurements can be used as an independent test set for these cities. We used ground measurement data from Accra and Hong Kong collected through previous spatial monitoring field campaigns. For Hong Kong, we used data from 97 NO_2_ sites and 52 PM_2.5_ sites collected in 2014 as detailed elsewhere [33]. For Accra, data was available from 35 NO_2_ sites and 32 PM_2.5_ sites collected from 2019 to 2020 [18], [34], [35].

### 2.4 Modelling approach

We used a ResNet [36] architecture where outputs are continuous pollutant levels (a regression task as opposed to classification) to train separate deep learning models for estimating NO_2_ and PM_2.5_ concentrations. We selected ResNet as it has a global average pooling at the end, which allowed us to generate post-hoc model visualizations highlighting most relevant areas in the image for estimates using class activation maps (CAMs) [37]. Specifically, we used the ResNet18 architecture with pre-trained weights on ImageNet available in PyTorch [38]. We added a fully connected layer at the end with a single output value suited for the regression task, replacing the soft-max layer after Layer 4. We fixed pre-trained weights up to Layer 4, and fine-tuned and trained weights for Layer 4 as well as the fully connected layer at the end (Figure S1).

We first evaluate intracity performances of image-based deep learning models for estimating air pollution. For labels, annual mean pollutant levels from the city-wide models were used for London, New York, and Vancouver. Networks were trained and tested on image-label pairs from the same city using four-fold cross validation. The results show how well models perform in capturing visual features associated with pollution levels at the local level.

Second, we explored how trained models transfer across cities. Transferability of LUR models across space was previously evaluated and found to perform poorly without local calibration [39]–[41]. In this study, the networks trained on combined data from two cities were tested on the held-out city. This process was repeated three times holding out a different city each time. The results show how well identified features associated with different pollution levels transfer across cities.

Third, we focused on the transferability of deep learning models to Accra and Hong Kong. The most appealing promise of using street imagery for air pollution estimation is its potential for scaling globally at low cost, especially to the very large number of cities which lack local air pollution measurements or models (e.g., cities from different countries, development trajectories, pollution sources, and climates) [7]. To test the viability of such an application, we selected Accra and Hong Kong as test cities where we had access to spatially distributed air pollution measurements collected through field campaigns. Note also that these settings differ geographically and with respect to development level, climate and pollution sources from all of the training cities.

The root-mean-squared-error (RMSE), normalized RMSE (NRMSE), mean error (ME), and Pearson correlation coefficient (r), and coefficient of determination (R^2^) were computed using hold-out sets for each of the cities and experiments. R^2^ value for predicted vs. observed concentration were used to allow for quantitative comparisons with earlier work that focused on intercity transferability of LUR models, with results ranging between R^2^ = 0.33 and 0.51 [41]. We generated estimation maps for each city to assess how well spatial variations were captured. Finally, we evaluated performance using ground monitoring stations data.

## 3 Results and Discussion

### 3.1 Intracity performance for London, Vancouver, and New York

Labelled images, separately for NO_2_ and PM_2.5_, were prepared for each of the three cities. Each of these images were matched with labels available from city-wide pollution rasters using the coordinate information for each image. We trained separate models for each city using four-fold cross validation. In each fold, 75% of data (i.e., randomly selected image-outcome pairs for 75% of grid points) were used for training the network and the remaining 25% were withheld. We then measured how well the trained network uses images to estimate outcomes at locations that were not used in training from the same city. We repeated this process four times holding out a different 25% of data each time.

Full results are available in Table 1. For NO_2_, intracity models achieved r values of 0.79, 0.87, and 0.73 (R^2^= 0.58, 0.75, and 0.52) respectively for London, New York, and Vancouver. Lower performances were observed for PM_2.5_ where r values were 0.79, 0.85, and 0.60 (R^2^= 0.59, 0.71, 0.33). This was expected and consistent with previous work, as local variation for NO_2_ is higher and influenced more by local (mobile) sources compared with PM_2.5_ that is influenced by regional levels and secondary production which is unlikely to be closely related to micro-scale spatial patterns of sources. Average RMSE values across cities were 4.3 µg/m^3^ for NO_2_ and 1.0 µg/m^3^ for PM_2.5_. Best performances were achieved for New York and poorest performance was observed for Vancouver particularly for areas of low pollution.

Figure 2 shows that the spatial patterns of pollution are better captured for NO_2_ compared to PM_2.5_ by image based deep learning models. Similar patterns over space are observed for both label and prediction maps even though predictions are only based on street level images, i.e., no spatial information or smoothing method was used for generating prediction maps. Image-based deep learning models identify major roads and distinguish them from smaller streets and associate them to higher levels of pollution concentration. City centers characterized by higher building and population density are also identified and predicted as high-pollution areas. These results are consistent with inputs to LUR and dispersion models used as labels described in Section 1.1. Prediction errors for all cities were particularly high for low pollution areas. Spatial patterns in errors reflect the impact of features such as geography (e.g., proximity to the coast and mountains in Vancouver). This may, in part, reflect that street level images are predominantly collected from streets by driving cars and availability is limited for low pollution areas such as parks, forests and coastal areas. The models use images only for predictions with no additional information on the spatial context (e.g., proximity to parks, mountains, ocean etc.). Data from other sources (e.g., satellite images, built environment datasets) can be used to provide information about the spatial context within a multi-model learning framework can help improve performances in the future [20].

**Figure 3:**
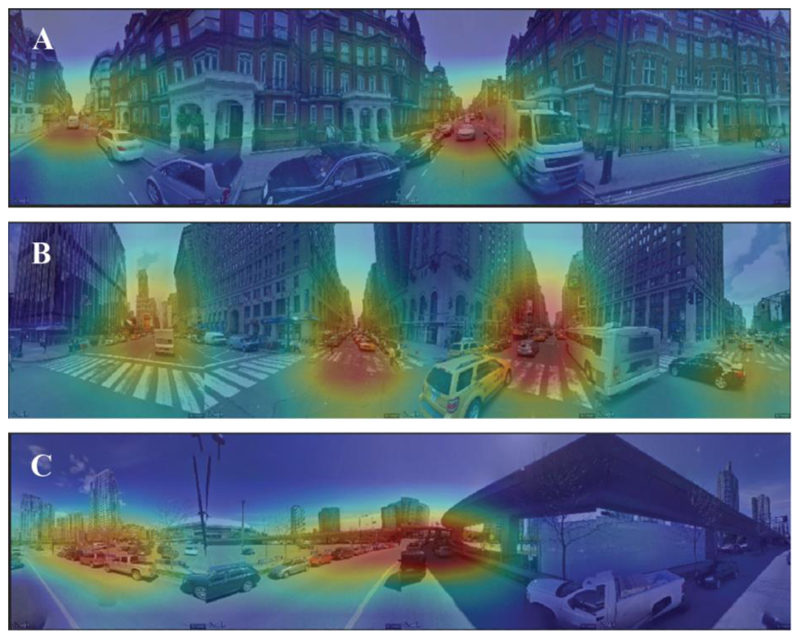
Examples of class activation maps from (A) London, (B) New York, and (C) Vancouver. These examples were selected from locations where both the label and prediction NO_2_ values were high as follows: 53.3 µg/m^3^ and 76 µg/m^3^ for (A), 20.8 µg/m^3^ and 16.7 µg/m^3^ for (B), 63.5 µg/m^3^ and 61.3 µg/m^3^ for (C). Red areas indicate parts of the image that the network considers as positively influencing pollution levels. The visualizations show the network identifies cars, roads, and high density building as major sources of pollution. More examples are available in SI that are less appealing and harder to interpret especially from areas of low- and mid- range pollution levels.

We used class activation maps (CAMs) for additional insights to image regions that are used by the model for making pollution predictions, [42], [43]. CAMs offer a post-hoc visualization method using the average pooling layer at the end of ResNet18. Figure 3 shows some examples from locations where both the label and predicted pollution values were very high from each city. The areas of the image that substantially contribute positively to the final prediction value are highlighted in red. We observe that roads, cars, and high building density is being highlighted in these images. Interpretation of CAM visualizations become harder and less appealing for areas having mid- or low-range pollution levels (see more examples in SI). This could be related to the general difficulty of interpretability of deep learning models and associated inadequacies in the proposed CAM methods but could also potentially be explained by the fact that no specific image region is identified as a major source of pollution, which also correctly results in lower pollution estimates.

We analyzed the association between the model estimates results and ground monitoring site measurements separately for each study city. Sites that had co-located model estimates within a distance of less than 50 m were included. It was not possible to compute r for PM_2.5_ in Vancouver as we only had two qualifying measurement sites. Correlations between estimated values and annual mean concentrations were high: NO_2_ models achieved r values of 0.90, 0.76, and 0.97 (R^2^ = 0.81, 0.58, 0.95, N = 8, 47, 4) respectively for London, New York, and Vancouver; PM_2.5_ models achieved r values of 0.72 for London and 0.86 for New York (R^2^ = 0.51, 0.74, N = 9, 4). The number of available monitoring stations for evaluations were limited, so results should be interpreted with caution. These values are comparable to performances of the underlying models whose outputs were used for training, suggesting that images can capture a substantial part of pollution sources often used as inputs for model for training. In our current setting, we are unable to generate daily or hourly estimates of pollution as we were restricted by image availability.

### 3.2 Intracity performance for London, Vancouver, and New York

For evaluating transferability of learning, we trained three separate models using three-fold cross validation. In each fold, combined data from two cities were used for training the network and the third city was withheld. We then measured how well the trained network uses images to predict outcomes in the holdout city not used for training. Resulting performance metrics can be compared with intracity results presented in the previous section. In regression tasks, it is well known that deep-learning models do not generalize well to previously unseen values. This was, by design, a specifically challenging setting as label data distributions used for the selected three cities were almost non-overlapping for NO_2_ and PM_2.5_ (Figure 1). For example, when training on combined NO_2_ data from Vancouver and New York, labels used in training were mostly ranged between 0 to 40 µg/m^3^. Accordingly, predictions were also in this range when making predictions in London. Yet, target data NO_2_ concentrations were much higher in London mostly ranging between 20 to 60 µg/m^3^ resulting in large RMSEs. As a result, estimation of absolute levels was poor. We also wanted to understand if there is a way to shift estimation distributions to match the target distributions to achieve lower levels of absolute error and whether our models were successful in capturing spatial variation and relative pollutant levels. For this purpose, we transform and adjust estimation distributions using the mean and standard deviation of the labels ($\bar{x}$ and *σ*_*x*_) from the target city using the following equation: $y\prime =\bar{x}+(y-\bar{y})*(\sigma_{x}/\sigma_{y})$ where *y*′ and *y* are, respectively, the adjusted and pre-adjusted estimation values. This post-hoc adjustment step influences RMSE and NRMSE values, but not r and R^2^values. In practice, some prior information on means and variation of air pollution levels from the target city will be required for this post-hoc adjustment step. We reported performances from the adjusted model estimates in Table 1 and Figure 2 (results from non-adjusted model estimates are provided in the SI).

When the network trained on two source cities was used to make predictions in the hold out target city, the performances were much lower. Pearson correlation between true (label) and predicted values were 0.42, 0.60, and 0.28 (R^2^ = 0.18, 0.36, 0.08) for London, New York, and Vancouver for NO_2_`, and 0.37, 0.47, and 0.06 for PM_2.5_ (R^2^ = 0.14, 0.22, 0). These correlations were lower, and the errors were larger, than those for intracity results (Section 2.1). Correlations between estimated values and annual mean concentrations from ground monitoring stations were also lower with r values ranging between 0.51 (R^2^ = 0.00, N=4) for Vancouver and New York (R^2^ = 0.26, N=47) to 0.82 (R^2^ = 0.67, N=8) in London for NO_2_, and 0.29 (R^2^ = 0.09, N=4) in New York and 0.46 (R^2^ = 0.22, N=9) in London for PM_2.5_. This is consistent with previous work where transferability of LUR models were evaluated [39]–[41] and found low performances for NO_2_. Specifically, previous work reported transferability performances of a LUR modeled trained on Vancouver was R^2^ = 0.51 (N=40) for Victoria (another city in Canada) and R^2^ = 0.33 (N=26) for Seattle (a city in another country). We observe similar performances from intercity NO_2_ models R^2^= 0.67 (N=8) for London, R^2^=0.26 for New York (N=47) and R^2^ = 0.00 (N=4) for Vancouver. We use less data input, yet our evaluation set is limited since we had limited numbers of available monitoring stations in London and Vancouver. Figure 2 shows that spatial variation for NO_2_ can be captured to a limited degree. It is, however, much harder to transfer across cities to estimate spatial variability of PM_2.5_. This likely results from differences in important PM_2.5_ sources between cities, in regional (e.g., topographic, geographic) influences on PM_2.5_ spatial patterns and on meteorological impacts on dispersion and secondary PM_2.5_ production. For example, Vancouver is both mountainous and with sea breeze influences that will not be identified by street level images, while New York City is downwind of industrial emissions sources and power generation facilities that were located outside of the imagery domain. Even though it is possible to transfer image-based deep learning models between cities, success will depend not only on visual similarity of features associated with pollution levels but also differences in absolute levels and distributions of pollution between source and target cities. Some of this information would be available from global models [44]. Further research is needed to investigate the most efficient approaches to local calibration to help produce models that are as predictive as their performances in training cities [41], [45].

Finally, we wanted to understand if image-based estimates also perform better in locations where local LUR and dispersion models had lower error. For a comparative analysis of image-based prediction errors and locally calibrated models (originally used as labels in training), we computed relative errors for each ground monitoring station location. The numbers of monitoring stations available for evaluations were, unfortunately, too low to generalize our findings. Still, as expected, for NO_2_, intracity image-based errors were highly correlated with local LUR/dispersion model errors with correlations of r=0.62 (N=8), 0.72 (N=47), 0.99 (N=4) for London, New York, and Vancouver. When transferring between cities for NO_2_, we got a much lower correlations for New York at 0.08 where we had the largest number of data points N=47. For other cities the correlations remained relatively high at r=0.60 for London and r = 0.98 for Vancouver. For PM_2.5_, within city correlations between relative errors from intracity image-based estimates were r=0.51 for London (N=9) and r=0.21 for New York (N=4); we could not include Vancouver with N=2. Intercity image-based transfer was even more difficult for PM_2.5_, in line with other findings, where the correlations were very low or in the wrong direction with r=0.35 and r=-0.77. These findings are in line with our main results where we showed that image-based models can learn local features when trained on image-label pairs from the same city. It is, however, much harder to transfer across cities.

### 3.3 Evaluation of transferability to Accra and Hong Kong

We evaluated how well a model trained on combined (pooled) data from all three source cities i.e., London, New York, and Vancouver, performed in estimating pollution levels in Accra and Hong Kong. Evaluation of transferability performance is difficult for cities where city-wide estimates of annual pollutant levels are scarce. Here, we used monitoring station data from field campaigns carried out for Hong Kong [33] and for Accra [18]. In Hong Kong, measurements were collected in two sampling campaigns during the warm and cool seasons. Roadside measurements of NO_2_ were made for 2-3 week periods with Ogawa passive samplers at 97 locations. PM_2.5_ was measured with the TSI SidePak personal aerosol monitors at 84 sites for 24 hrs. Both the NO_2_ and PM_2.5_ measurements at the above sites were adjusted for day-to-day variation with continuous measurements made at a subset of four monitoring sites. A similar protocol was employed in Accra (10 yearlong sites and 136 weeklong sites). Sites with street level images available within a 50m distance threshold were included for evaluations in Table 1.

**Figure 4:**
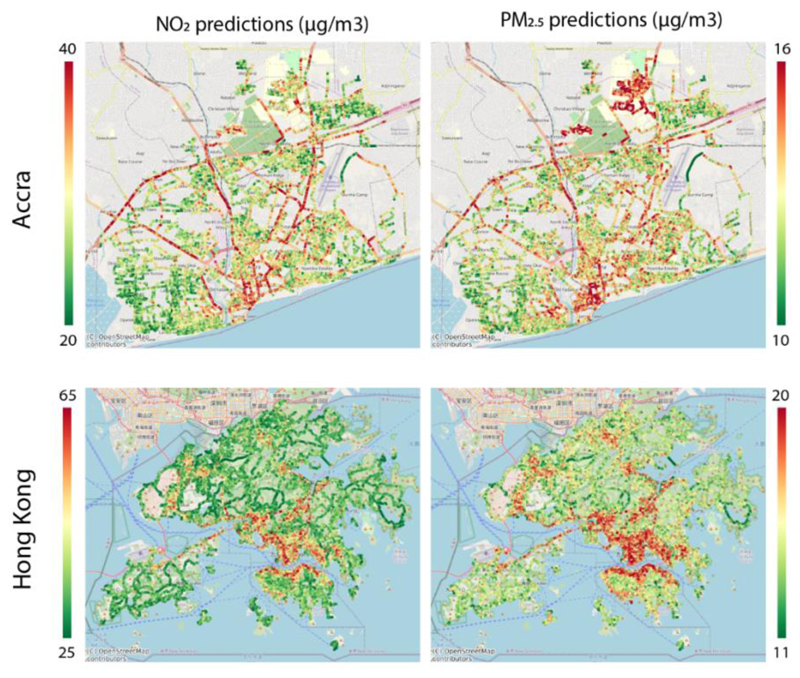
Prediction maps for NO_2_ and PM_2.5_ for Accra and Hong Kong. Prediction maps of mean NO_2_ and PM_2.5_ levels for Accra and Hong Kong. Models were trained on combined data from London, New York, and Vancouver and tested on images for Accra and Hong Kong. We adjusted the color bar scale to visualize the spatial patterns for each city and pollutant where red areas correspond to high pollution and green to low pollution.

Estimation maps shown in Figure 4 associate major roads and city centers with high pollution. However, when we evaluate performances with ground monitoring stations, the performances were poor (r = 0.46, R^2^ = 0.21 (Hong Kong) and 0.21, R^2^ = 0.06 (Accra) for NO_2_ in and, r = 0.02, R^2^ = 0.02 (Hong Kong) and 0.12, R^2^ = 0.01 (Accra) for PM_2.5_). In Hong Kong, higher pollution levels near China, as expected, also are not captured as the images do not contain information on proximity to high pollution regions. The city center, however, is successfully identified as a relatively high pollution area.

## 4 Conclusions

The focus of this work was to understand whether and to what extent information contained in street level images only, with limited no or limited information on the local conditions, can predict spatial variation in annual pollution levels. Performances of image-based models, when trained and tested on the same city, for estimating mean annual levels of air pollution were high. Model performances of these deep learning models using only images as input were comparable to performances of traditional models that require substantially higher levels input data. This suggests that images can capture a substantial part of pollution sources often used as inputs for model for training along with additional insights from post-hoc visualizations highlighting image regions that are used by these models.

Like LUR models, transferability of models between cities in different geographies is more difficult. Specifically, we found that transferability to Hong Kong and Accra was much more difficult than transferability between London, Vancouver, and New York. Image-based estimation models work better when transferring between cities that share similar source of pollution. It is however a much larger challenge to transfer models calibrated in cities with fundamentally different sources of pollution (e.g., type of cars, biomass burning [46]). In other words, models’ ability is dependent on the similarity of underlying sources of pollution that can be captured by images only. Hence, their ability to scale up to global coverage is also dependent on availability of label data from cities with similar source profiles.

Our findings suggest several open questions for future work. Would locally calibrated LUR model outputs, where and when available, for a city like Accra transfer well to other West African cities (e.g., in Nigeria) with similar sources of pollution? Would images based only models transfer well to cities within the same country and over time? Can active learning approaches of machine learning be utilized for effective local calibration where the measurement points in target cities are sample in an optimal way? Can images be combined with other globally available local sources of information (e.g., satellite-based air quality estimates, aerial images satellite data on proximity to parks, oceans, roads, OpenStreetMaps) to make better predictions for scaling up globally? How can we modify data collection protocols to better utilize images and image-based predictions? Can daily or hourly estimates be generated if images are available at high temporal resolution? We hope that future research, based on this study, can help address these questions and enable better models of pollutant levels with global coverage to support global efforts to reduce pollutant levels and establish health effects.

## Supplementary Material

Supplementary Material

## Figures and Tables

**Table 1:** Summary of model performances for all experiments

**City-wide LUR based estimates of air pollution**															
	**London**					**New York**					**Vancouver**				
	*r*	RMSE	NRMSE	R^2^	ME	*r*	RMSE	NRMSE	R^2^	ME	*r*	RMSE	NRMSE	R^2^	ME
NO_2_	*N = 94714*					*N = 49575*					*N = 60562*				
Intracity	0.79	7.31	0.20	0.62	-0.19	0.87	3.83	0.18	0.75	0.34	0.73	1.81	0.19	0.52	0.11
Intercity	0.42	21.38	0.58	0.18	18.80	0.60	12.83	0.59	0.36	-8.66	0.28	17.57	1.81	0.08	-16.69
Adj. intercity		12.07	0.32		0.00		6.89	0.31		0.00		3.12	0.32		0.00
PM_2.5_			
Intracity	0.79	0.88	0.06	0.59	0.04	0.85	0.61	0.06	0.71	0.069	0.60	1.63	0.49	0.33	0.14
Intercity	0.37	5.77	0.40	0.14	5.52	0.47	4.06	0.42	0.22	-2.21	0.06	9.10	2.73	0.00	-8.80
Adj. intercity		1.55	0.11		0.00		1.17	0.12		0.00		2.75	0.82		0.00
**Ground monitoring stations**															
	**London**					**New York**					**Vancouver**				
	*r*	RMSE	NRMSE	R^2^	ME	*r*	RMSE	NRMSE	R^2^	ME	*r*	RMSE	NRMSE	R^2^	ME
NO_2_	*N = 8*					*N = 47*					*N = 4*				
Intracity	0.90	17.71	0.27	0.81	13.47	0.76	27.97	0.49	0.58	26.29	0.97	2.63	0.24	0.95	1.65
Intercity	0.82	46.22	0.71	0.67	41.91	0.51	22.30	0.39	0.26	17.90	0.51	13.70	1.23	0.00	-12.14
Adj. intercity		13.52	0.21		0.00		14.38	0.25		0.00		4.81	0.43		0.00
PM_2.5_	*N = 9*					*N = 4*					*N = 2*				
Intracity	0.72	2.19	0.13	0.51	-0.34	0.86	1.15	0.10	0.74	1.56	-	1.04	0.25	-	0.70
Intercity	0.46	8.03	0.48	0.22	7.54	0.29	5.26	0.47	0.09	-4.41	-	8.81	2.15	-	-8.7
Adj. intercity		2.72	0.16		0.00		0.65	0.06		0.00		0.78	0.19		0.00
	**Hong Kong**					**Accra**				
	*r*	RMSE	NRMSE	R^2^	ME	*r*	RMSE	NRMSE	R^2^	ME
NO_2_	*N = 97*					*N = 35*				
Intercity	0.46	63.30	0.58	0.21	51.63	0.25	38.55	0.63	0.06	30.19
Adj. intercity		42.42	0.39		0.00		28.65	0.47		0.00
PM_2.5_	*N = 52*					*N = 32*				
Intercity	0.02	8.35	0.36	0.00	3.90	-0.12	13.02	0.61	0.01	8.09
Adj. intercity		9.76	0.42		0.00		11.91	0.56		0.00

* ME and RMSE values are reported in µg/m3.

## Data Availability

All datasets used in this paper are publicly available and the URLs are provided in the Data section. Upon publication, the code will be available at https://github.com/esrasuel/sview-pollution.
