# Supplementary material for: What you see is what you breathe? Estimating air pollution spatial variation using street level imagery
Source: Remote Sens (Basel). Author manuscript; Available in PMC 2023 Sep 16. (PMC7615101; doi:10.3390/rs14143429)
Supplement: Supplementary Material [file EMS187696-supplement-Supplementary_Material.pdf]

Supporting Information

What You See Is What You Breathe? Estimating Air Pollution Spatial Variation Using Street-Level Imagery

Esra Suel*, Meytar Sorek-Hamer, Izabela Moise, Michael von Pohl, Adwait Sahasrabhojanee, Ata Akbari Asanjan, Raphael E. Arku, Abosede S. Alli, Benjamin Barratt, Sierra N. Clark, Ariane Middel, Emily Deardorff, Violet Lingenfelter, Nikunj C. Oza, Nishant Yadav, Majid Ezzati and Michael Brauer

* Corresponding author, e-mail: esra.suel@imperial.ac.uk

This PDF file includes:

- Description of additional CAM examples
- Stringent segmentation for intracity experiments
- Figure S1 to S4

Description of additional CAM examples

Below, we present additional examples of class activation maps from (A) London, (B) New York, and (C) Vancouver where models were trained and tested on the same city. These examples were selected from locations where Fig.S1. label NO2 values were in the highest decile, but predictions were in lower deciles, Fig.S2. label NO2 values were in the lowest decile but predictions were in higher deciles, and Fig.S3. label and prediction NO2 values were both in the lowest decile.

The visualizations are much less appealing compared to the examples given in Figure 3 in main text where both prediction and label NO2 values were in the highest decile. This may be related to relatively lower performances of deep learning models in making predictions in lower pollution areas where the networks cannot properly identify sources of pollution. Instead, what is visible from these images is that they often have a similar pattern, where the bottom pixels corresponding to road are highlighted in red that are contributing positively to the final prediction value, and the top pixels corresponding to skies, homes, and trees are highlighted in blue that contribute negatively to the prediction value. For Vancouver, the resulting images are sometimes counterintuitive which is consistent with low prediction performances achieved, where it is the trees and skies that are highlighted in red (suggesting positive contribution to the predicted pollution level) and roads in blue (suggesting negative contribution to predicted pollution level).

Stringent segmentation for intracity experiments

In the main paper, the evaluation of intracity performances were based on four-fold cross validation where the hold out samples were drawn randomly. For a more stringent segmentation approach for train-test splits, we conducted additional experiments where we

excluded larger sections, based on Middle Super Output Area (MSOA) definitions, for London when we create cross-validation train-test splits for NO_2. MSOAs are designed for reporting small area statistics in England and Wales with an average population of 8,346 in London in 2010[1]. While it is possible to do more experiments with even larger sections of the city to be excluded, without a boundary definition it becomes harder to systematically divide up the city to blocks while also maintaining a balanced training set where we can ensure images from different deciles are well-represented both in training and test splits. We view intercity experiments are the extreme case (the harder test), where the task beyond transfer between different areas in one city, and to transfer across cities from different countries. As expected, these performances are very similar but slightly lower than our initial random draws, yet much better compared to intercity performances as shown in Table S1.

Table S1: Comparison of intracity performances using random train-test splits vs. a more stringent approach using Middle Super Output Areas in London.

	**City-wide LUR based estimates of air pollution**				
	**London**				
	r	RMSE	NRMSE	R^2	ME
**NO_2**	$N = 94714$				
Intracity	0.79	7.31	0.20	0.62	-0.19
Intracity (stringent segmentation)	0.78	7.46	0.20	0.61	0.02

[1] https://data.london.gov.uk/dataset/msoa-atlas

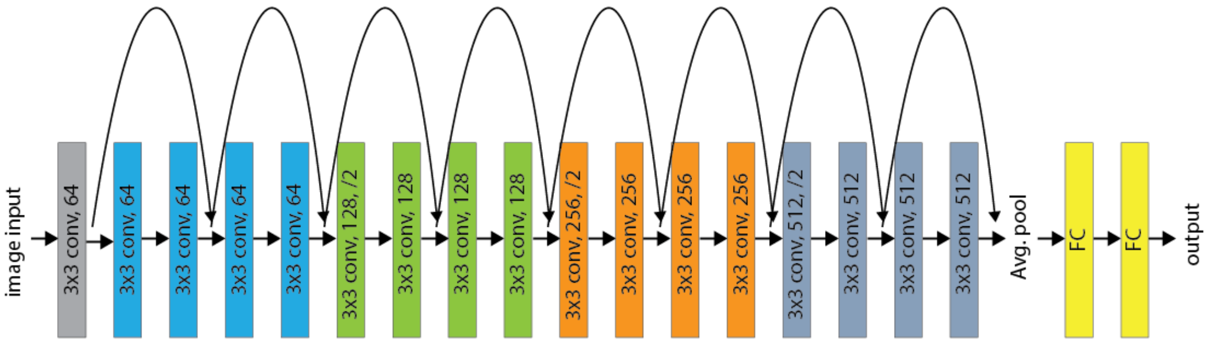

Figure S1: Modified ResNet18 architecture used for all experiments.

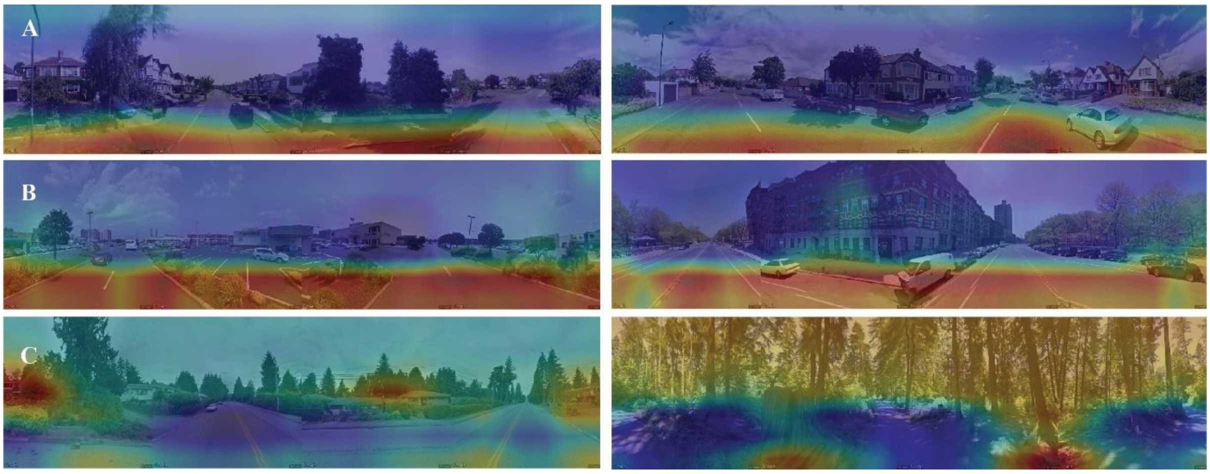

Figure S2: Example images where label NO2 values were in the highest decile, but predictions were in lower deciles.

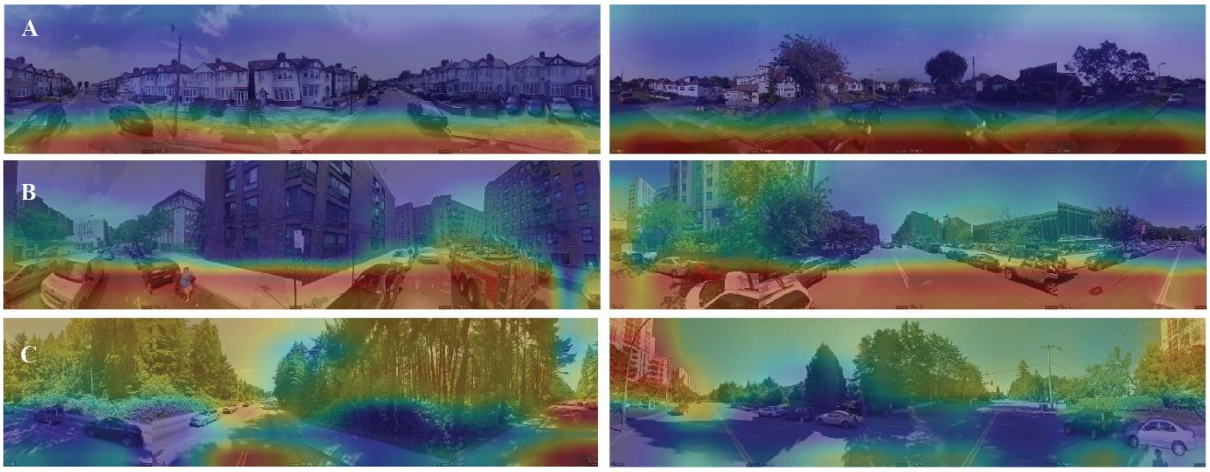

Figure S3: Example images where label NO2 values were in the lowest decile, but predictions were in higher deciles.

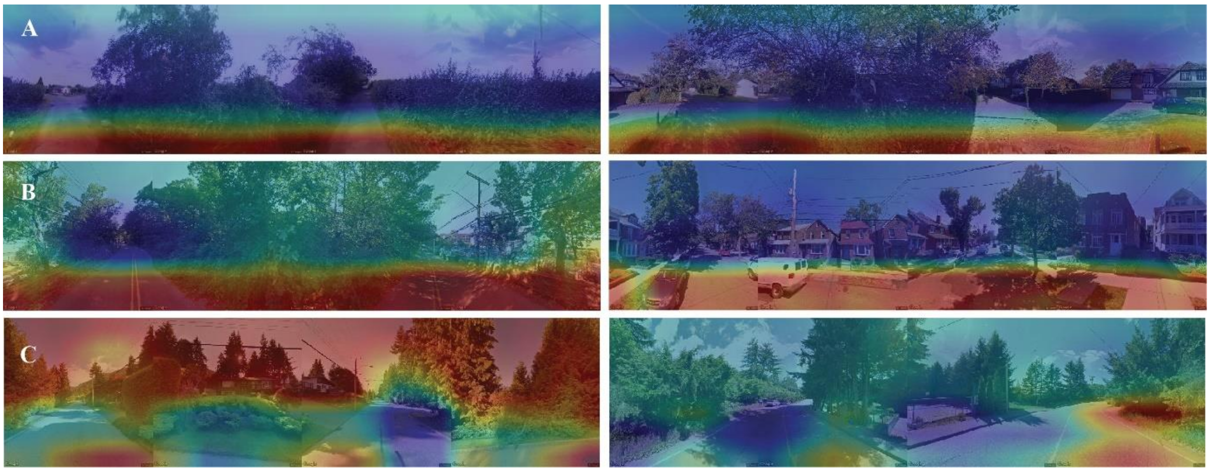

Figure S4: Example images where both label and prediction NO2 values were in the lowest decile.
